# The double-edged sword of TOX: orchestrating T cell exhaustion and epigenetic remodeling in the tumor microenvironment

**DOI:** 10.3389/fimmu.2026.1957165

**Published:** 2026-10-13

**Authors:** Gaia Zuccolotto, Lodovica Borghese, Alessia Rebuli, Antonio Rosato, Anna Tosi

**Affiliations:** 1 Immunology and Molecular Oncology Diagnostics, Veneto Institute of Oncology IOV-IRCCS, Padova, Italy; 2 Department of Surgery, Oncology and Gastroenterology, University of Padua, Padova, Italy

**Keywords:** cancer, epigenetics (chromatin remodeling), T cell exhaustion, TOX, tumor microenvironment

## Abstract

The capacity to profoundly change chromatin domains and in turn drive or inhibit gene expression makes chromatin modulators a powerful tool adopted to regulate crucial steps during development and cell lineage specification. These proteins can however be as well engaged to irreversibly reprogram cells toward pathological outcomes such as cancer. An example of such chromatin remodelers is the family of Thymocyte-selection associated high mobility box proteins (TOX), which has rather pleiotropic functions during development and in cancer. In light of the most recent findings, this review gives an overview on the many different roles played by TOX family of proteins in physiological processes, and of the context-dependent effects that TOX induces in a variety of tumors. A particular attention is given to the contribution of TOX to the mechanism of T cell-exhaustion and to the “exhaustion lock” - no longer viewed as a dysfunctional immunological program but rather as a sophisticated strategy evolved by the immune system to keep control during events of chronic stress. The therapeutic potentials of TOX as valuable biomarker and target in cancer and immunotherapy as well as in adoptive cell therapies are also discussed.

## 1 Introduction

The orchestration of a robust immune response against malignancies relies heavily on the functional persistence of tumor-infiltrating lymphocytes (TILs). However, within the immunosuppressive tumor microenvironment (TME), continuous exposure to tumor antigens and chronic T-cell receptor (TCR) signaling triggers a state of functional impairment known as T cell-exhaustion (1). Far from a random cellular failure, exhaustion represents a coordinated, evolutionary adaptation designed to mitigate extensive tissue damage and immunopathology under persistent inflammatory conditions (2). In oncology, however, this “low-power mode” acts as a critical bottleneck, driving tumorigenesis, permitting immune evasion, and serving as a major determinant of resistance to standard treatments and immune checkpoint blockade (ICB).

At the center of this profound transcriptional and epigenetic metamorphosis is Thymocyte Selection-Associated High-Mobility Group Box Protein (TOX). Originally discovered as a transiently induced nuclear regulator governing thymocyte selection and the lineage commitment of adaptive and innate lymphoid populations, TOX has emerged as the master architect of the exhausted T cell-landscape (3).

By comprehensively evaluating TOX foundational contribution to physiological lymphopoiesis, its dual role in inflammation, by promoting effector function in CD4^+^ T cells, while driving T cell-exhaustion in CD8^+^ T cells, and finally its pathological subversion in solid and hematologic tumors, this review aims to consolidate our understanding of the “exhaustion lock”. Unravelling the molecular mechanisms, lineage dualities, and clinical associations of TOX will provide vital insights needed to design effective combinatorial immunotherapies, optimize adoptive cell engineering, and ultimately breach the epigenetic barriers that limit durable clinical responses in cancer.

## 2 The TOX protein family: structural features and functional diversity

Structurally, TOX is a 526-amino acid protein organized into three functionally distinct regions: an acidic N-terminal domain, a bipartite nuclear localization signal, and a centrally located High-Mobility Group (HMG)-box DNA-binding motif (3, 4). The HMG-box adopts a canonical three-helix L-shaped fold that engages the DNA minor groove in a sequence-independent manner (4, 5). Although most characteristics of TOX are similar to other HMG-box proteins, it lacks a key hydrophobic wedge residue that disables the induction of DNA bending (6). TOX has been proposed to represent a functionally distinct class within the HMG-box protein superfamily, mediating its functions through direct protein-protein interactions via its HMG-box domain rather than through DNA binding (7). In the context of T-cell Acute Lymphoblastic Leukemia (T-ALL), Lobbardi and colleagues demonstrated that TOX binds directly to the Ku70/Ku80 heterodimer, the initiating complex of the non-homologous end joining (NHEJ) DNA repair pathway, and inhibits its recruitment to double-strand break sites, thereby promoting genomic instability (7).

Whether analogous protein-protein interaction mechanisms operate in other cellular contexts in which TOX has been implicated, including its roles in lymphocyte development and CD8^+^ T cell exhaustion, remains to be established.

Consistent with a specialized rather than housekeeping function, TOX displays a tightly restricted expression profile, with predominant expression in the thymus and substantially lower levels in liver and brain (3, 4).

TOX belongs to a gene family comprising four conserved paralogs, TOX1, TOX2, TOX3, and TOX4, located on distinct chromosomes in both mice and humans, and all sharing the HMG-box domain (**Table 1**). Outside this region, however, their N-terminal and C-terminal sequences diverge substantially, consistent with partially non-overlapping biological functions (4, 6). TOX1, hereafter referred to as TOX, is the founding and most extensively characterized member of the family. Initially identified as a regulator of thymocyte selection (3), subsequent work revealed a far broader portfolio: CD4^+^T cell lineage specification, regulatory and invariant Natural Killer (NK) T cell development, NK cell maturation, lymphoid tissue inducer cell generation, and innate lymphoid cell progenitor specification (**Figure 1**). Collectively, these findings establish TOX as a pleiotropic regulator operating at multiple lymphoid commitment checkpoints (8–10). TOX has also emerged as a central transcriptional regulator of CD8^+^ T cell exhaustion in chronic infection and cancer, and it is increasingly investigated as a candidate biomarker in oncology (11). Notably, its function is not confined to enforcing an exhaustion program. Indeed, a recent work by Naizir et al. demonstrates that in CD4^+^ T cells, TOX is required for CD4^+^ T helper 1 (T_H_1) differentiation and IFN-γ production, and is essential for the generation of IFN-γ–producing T follicular helper (T_FH_) 1 cells during infection, highlighting a context-dependent and lineage-specific role (12).

**Table 1:** Physiological expression patterns and functions of TOX paralogs.

Paralogs	Primary biological functions
TOX	-Thymocyte selection -Lymphoid lineage commitment -CD8^+^ T cell exhaustion in chronic infection and cancer -CD4^+^ Th1 cell differentiation -IFN-γ production in CD4^+^ Th1 cells -IFN-γ producing T_FH_ cell generation
TOX2	-NK cell development -CD4^+^ T_FH_ cell development -CD8^+^ T cell exhaustion
TOX3	-Ca^2+^ responsive neuronal survival regulation in the brain -ER target gene transcriptional regulation in mammary luminal epithelium
TOX4	-Regulation of RNA polymerase II-mediated transcription -DNA double-strand break repair via NHEJ

See details in text.

**Figure 1:**
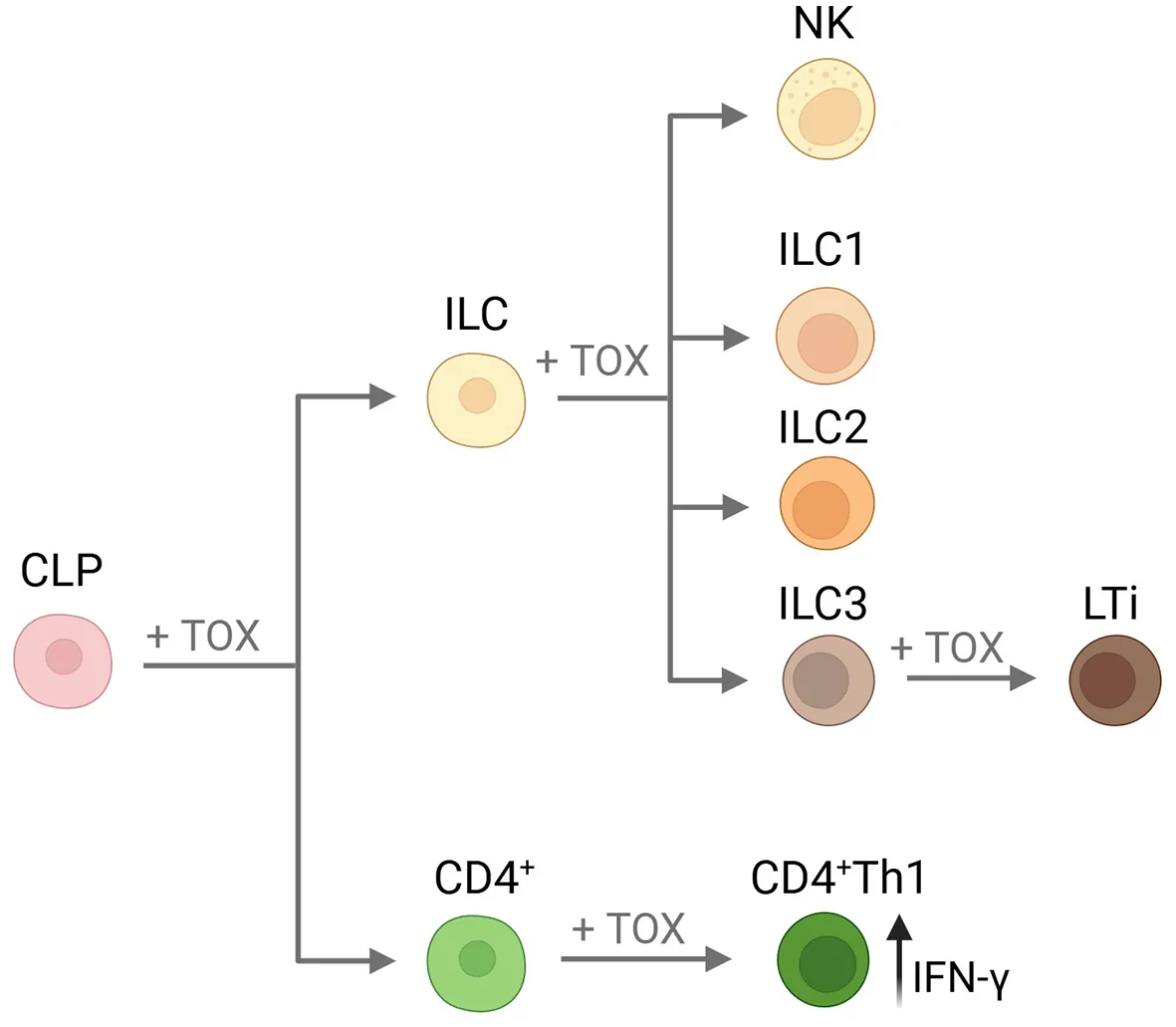
Physiological role of TOX in CD4^+^ T cell and lymphoid development. Diagram of lymphoid development and CD4^+^ T cell differentiation. + Tox indicates stages at which activation of TOX is required. For details see text. CLP, Common Lymphoid Progenitor; ILC, Innate Lymphoid Cell; NK, Natural Killer cell; LTi, Lymphoid Tissue inducer; CD4^+^, CD4^+^ T cell; CD4^+^Th1, CD4^+^ T cell with Th1 effector function; IFN-γ, Interferon gamma. (Image generated with BioRender).

The remaining members of the TOX family fulfils distinct biological roles. TOX2 cooperates with TOX1 as a downstream transcriptional target of the calcineurin-Nuclear Factor of Activated T cell (NFAT) axis in driving CD8^+^ T cell exhaustion (13), and independently governs NK cell and T_FH_ cell development via regulating chromatin accessibility (14, 15). In T cell Acute Lymphoblastic Leukemia (T-ALL), nuclear TOX and TOX2 jointly assemble a repressive complex with the transcriptional corepressor LCOR and the deacetylase HDAC3 to suppress T cell immunoglobulin and mucin-domain containing-3 (TIM-3) expression, with leukemogenesis linked to pathological nuclear retention of TOX2 (16). TOX2 has also been identified as a super-enhancer-driven oncogene in extranodal NK/T cell lymphoma (17). TOX3, predominantly expressed in the brain, functions as a calcium-responsive neuronal survival factor, modulating Ca^2+^-dependent gene expression through interaction with the CREB–CBP complex (18, 19). The 16q12.1 locus harboring TOX3 has been identified as a breast cancer susceptibility locus by Genome-Wide Association Study (GWAS), with expression carrying divergent prognostic implications across tumor types (20, 21). Finally, TOX4 acts as a regulatory subunit of the PP1 phosphatase complex (PTW/PP1), controlling RNA Polymerase II C-terminal domain dephosphorylation and thereby transcriptional pausing, elongation and reinitiation (22). TOX4 further participates in DNA double-strand break repair via NHEJ, associating with the DNA-dependent protein kinase (DNA-PK) complex to support DNA-PKs activation and coordinating Phosphatase Nuclear Targeting Subunit (PNUTS) recruitment; its depletion impairs NHEJ efficiency and sensitizes tumor cells to genotoxic damage (23).

## 3 TOX expression during physiological development

### 3.1 TOX in innate lymphoid cell development: from progenitors to natural killer cells

Innate lymphoid cells (ILCs) comprise a family of lymphocytes lacking T cell receptor (TCR) and B cell receptor (BCR) expression, encompassing NK cells, ILC1, ILC2, ILC3, and the specialized lymphoid tissue inducer (LTi) subset. TOX acts as a primary gatekeeper during the initial stages of innate lymphopoiesis, governing the *in vivo* differentiation of common lymphoid progenitors (CLPs) into ILC-restricted lineages (10, **Figure 1**). Specifically, TOX deficiency resulted in early defects in the survival or proliferation of progenitor cells, as well as impaired ILC differentiation at a later stage (10), positioning TOX as a key determinant of early specification to the ILC lineage. This requirement extends across lineages: TOX deficiency imposed a cell-intrinsic block in the generation of ILC1 cells and functional ILC2 cells (10). Both NK cell development and lymphoid tissue organogenesis were inhibited in the absence of TOX, and the development of LTi cells, a rare subset with an integral role in lymphoid tissue organogenesis, was found to require TOX (9). As a consequence, *Tox*^^-^/^-^^ mice completely lack peripheral lymph nodes and show a severe reduction in the frequency and size of Peyer’s patches (9). While TOX drives early pan-ILC development, its regulation of the NK cell lineage is highly localized to specific maturation windows. Physiologically, Tox expression is significantly upregulated in immature NK cells within the bone marrow. This requirement is mirrored in human translational models: in *in vitro* systems tracking human NK cell differentiation from cord blood-derived CD34^+^ hematopoietic stem cells, TOX knockdown leads to a marked contraction of the emerging NK cell pool (24). Mechanistically, this process relies on a direct transcriptional axis that operates through a strict, stage-restricted window. At the NK progenitor (NKp) stage, TOX maintains the expression of Mst1, an intermediate kinase in the Hippo signaling pathway, with ChIP-seq data indicating a direct binding site at the Stk4 locus, and deletion of TOX specifically at the NKp stage severely impairs NK cell immune surveillance, accompanied by downregulation of IFN-γ and CD107a expression; conditional deletion of *Mst1* at the NKp stage recapitulates this TOX-deficient phenotype (25). Importantly, this genetic dependency is strictly temporal. Once cells successfully transition past these early developmental checkpoints, TOX becomes entirely dispensable for the maintenance, survival, and ongoing effector functions of mature NK cells, confirming a highly stage-restricted requirement in innate immunity (25).

### 3.2 β-selection

TOX expression is transiently induced during β-selection. Wilkinson and colleagues showed that TOX protein is upregulated in Double Negative 3 (DN3) thymocytes, a developmental stage lacking CD4 and CD8 expression and undergoing pre-TCR-mediated β-selection, and declines in late DN4 cells, a kinetic pattern consistent with transient induction by pre-TCR signaling rather than sustained expression (3). The functional significance of this wave was subsequently explored by Aliahmad and colleagues, who demonstrated that enforced *Tox* expression at the DN stage is sufficient to drive upregulation of both CD4 and CD8αβ co-receptors and to induce *CD8* locus demethylation, molecular events associated with successful β-selection, even in the absence of pre-TCR signaling (26). Consistent with a dispensable rather than obligatory role at this stage, *Tox*^^-^/^-^^ mice maintain normal frequencies of all four DN subsets, indicating that TOX is not required for the β-selection checkpoint (8).

### 3.3 Positive selection

Following β-selection, TOX is re-induced at the CD4^+^CD8^+^ double-positive (DP) stage through a mechanistically distinct upstream signal: calcineurin/NFAT-dependent TCR activation. This was demonstrated by the abrogation of TOX upregulation at the DP stage upon calcineurin inhibition and its rescue by a constitutively active calcineurin transgene (8). The functional consequences of this second induction wave are substantial: enforced TOX expression biases positive selection toward the CD8 lineage, inducing *Runx3* upregulation and *Cd4* silencing even in MHC-deficient mice (26). Nevertheless, the CD8^+^ single-positive (SP) cells generated under these conditions fail to mature fully or to leave the thymus, indicating that TOX recapitulates only a component of the TCR instructive signal and cannot substitute for it entirely (8, 26). Collectively, these findings position TOX as a nuclear effector that translates two subsequent and mechanistically distinct checkpoint signals, pre-TCR engagement at β-selection and calcineurin/NFAT activation at positive selection, into coordinated changes in co-receptor expression and lineage-associated chromatin architecture.

### 3.4 CD4^+^ T cell lineage commitment and specification

TOX is essential for progression through the CD4^lo^CD8^lo^ transitional stage that follows positive selection: in its absence, thymocytes fail to generate CD4^+^ SP cells, while CD8^+^ SP numbers and overall thymic cellularity remain largely intact, despite normal TCR-proximal signaling (calcium flux and ERK activation are unaffected, 8). This lineage-selective requirement places TOX at the core of CD4^+^ commitment itself, rather than as a general checkpoint for thymocyte survival.

The relationship between TOX and T helper-inducing POZ/Kruppel-like factor (ThPOK), the master CD4^+^ commitment factor encoded by *Zbtb7b*, was directly tested by enforcing ThPOK expression in *Tox*-deficient backgrounds. ThPOK efficiently repressed the CD8 lineage fate. The rescued cells, however, displayed an anomalous CD4^lo^ SP phenotype with persistently reduced *Cd4* transcription, and remained deficient in Id2, Foxo1, and endogenous ThPOK. TOX therefore establishes the CD4^+^ lineage gene program independently of, and in parallel to, ThPOK-mediated CD8 fate repression, a requirement that cannot be reduced to *Thpok* induction alone, even though TOX also contributes to *Thpok* locus activation (27).

Mechanistically, TOX operates at a regulatory node downstream of GATA3 and upstream of Thpok, integrating TCR-derived signals into the chromatin landscape of the CD4^+^ program. A key downstream effector is inhibitor of DNA binding 2 (Id2): TOX drives Id2 upregulation at the CD4^+^CD8^lo^ stage, which in turn relieves E protein-mediated repression and permits induction of Foxo1 and the broader set of CD4-lineage maturation genes — a step that ThPOK expression alone cannot restore (27). This TOX–Id2 axis is not unique to T cells: the same relationship operates in NK cells and ILC progenitors, pointing to a shared chromatin-based mechanism by which TOX coordinates early lineage specification across adaptive and innate lymphoid compartments (9, 10), (**Figure 1**). The consequences of this incomplete program extend into the periphery. CD4^+^ T cells generated in ThPOK-transgenic Tox-deficient mice fail to induce CD40L upon activation and show reduced FOXO1-dependent expression of Klf2, S1pr1, CCR7, and CD62L (27), indicating that TOX-dependent chromatin remodeling initiated during thymic development is required not only for lineage choice but for the acquisition of full CD4^+^ effector competence later in life.

## 4 TOX in CD4^+^ Th1 effector function and antitumor immunity

Beyond its developmental role, TOX has emerged as a driver of Th1 effector function in peripheral CD4^+^ T cells (**Figure 1**). CD4^+^ Th1 cells are defined by their production of IFN-γ and their role in orchestrating type 1 immune responses, including the activation of macrophages and the support of CD8^+^ T cell-mediated cytotoxicity (28). Canonically, their differentiation is driven by the transcription factor T-box expressed in T cells (T-bet, 29). In a recent study, Naizir and colleagues showed that TOX promotes IFN-γ production in CD4^+^ Th1 cells in a dose-dependent, T-bet-independent manner. This suggests that it acts through a parallel transcriptional axis rather than simply reinforcing the canonical Th1 program. TOX has been proposed to function as a master regulator of Th1 polarization by shaping chromatin accessibility at *loci* controlled by E26 Transformation-specific (ETS) transcription factors, again independently of T-bet (12).

This effector function has direct implications for antitumor immunity: IFN-γ activates M1-like macrophages, upregulates MHC-II on tumor cells to enhance antigen presentation, and promotes the recruitment of cytotoxic CD8^+^ T cells (30). Through these downstream effects, TOX-driven Th1 polarization positions TOX as a potential contributor to productive antitumor responses, in clear contrast to its role as a marker of dysfunction in exhausted CD8^+^ T cells.

Clinical data are consistent with this functional distinction, although the available human evidence remains correlative. Re-analysis of publicly available single-cell RNA-sequencing datasets showed that in early-stage hepatocellular carcinoma patients treated with neoadjuvant anti-PD-1 (31), TOX expression was enriched within a CXCL13^+^ CD4^+^ T cell population. Furthermore, the proportion of TOX-expressing CD4^+^ T cells was higher in responders than in non-responders (12). A comparable pattern emerged from re-analysis of a bladder cancer dataset (32), in which markedly higher TOX expression was found in a CXCL13-expressing CD4^+^ T cell subset (Naizir et al., 2026).

These observations are supported by independent single-cell studies, which converge on a CD4^+^ PD-1^+^ CXCL13^+^ population, commonly described as T_FH_-like, as a recurrent correlate of checkpoint-blockade benefit. A clonally expanded CD4^+^ PD-1^+^ CXCL13^+^ subset was identified in non-small-cell lung cancer (NSCLC) as the principal T cell partner of intratumoral antigen-presenting cells (33). Priming of this population in tumor-draining lymph nodes was required for an effective response to anti-PD-1 therapy (33). In melanoma, neoantigen-reactive CXCL13^+^ CD4^+^ T cells segregate into a T_FH_-like/memory cluster and a distinct cytolytic cluster (34). The intratumoral frequency of this compartment correlates with CD8^+^ T cell and macrophage activation, B cell maturation, and patient survival, with comparable associations reported in an independent breast cancer cohort (34). Ectopic expression of TOX, acting together with BATF, IRF4 and JunB at specific regulatory elements, is sufficient to induce *Pdcd1* alongside the chemokine/trafficking genes *Ccl3*, *Ccl4* and *Xcl1* (35), a transcriptional signature that overlaps with that of T_FH_-like CD4^+^ T cells in the tumor microenvironment. Whether TOX drives the CXCL13^+^ PD-1^+^ effector program of intratumoral T_FH_-like CD4^+^ T cells through this same BATF/IRF4/JunB-cooperative circuitry, and whether this contributes causally to checkpoint-blockade responsiveness, has not yet been tested directly and represents ground for further analysis. Collectively, these findings support a broader role for TOX-expressing T_FH_-like CD4^+^ T cells across tumor types beyond the function in lymphoid development and T cell exhaustion.

## 5 The Role of TOX during exhaustion of CD8^+^ T cells

### 5.1 From chronic stimulation to the “Exhaustion lock”

Cellular exhaustion is redefined not as a functional failure, but as a sophisticated evolutionary safeguard; it serves as a reminder that in biology, processes rarely occur by chance, and even the attenuation of a T cell power is orchestrated to prioritize the survival of the organism as a whole (36,, 37). At the heart of this transformation lies TOX (11). While T-cell activation is typically viewed through the lens of acute response, the persistence of tumor antigens rewires the signaling machinery, appointing TOX as the master architect of the “exhausted” epigenetic landscape (38). Indeed, the induction of TOX by chronic stimulation serves an evolutionary purpose preventing immunopathology (39). If T cells maintained peak effector functions (such as massive cytokine release and uncontrolled cytotoxicity) under conditions of permanent antigen exposure, the resulting systemic inflammation would cause catastrophic tissue damage (2). By inducing TOX, the immune system engages a molecular “lock”. This lock ensures that T cells survive the chronic encounter by entering a state of metabolic and functional “low-power mode”, preventing Activation-Induced Cell Death (AICD), while simultaneously protecting host tissues from uncontrolled T cell-mediated destruction (40,, 41).

Unlike acute infections, where naïve CD8^+^ T cells differentiate into highly functional effector CD8^+^ T cells (T_eff_) and then in long-lived memory CD8^+^ T cells (T_mem_, 42), the persistent presence of tumor antigens creates a state of chronic T cell receptor (TCR) signaling, forcing T cells into a specialized differentiation program known as exhaustion (**Figure 2**). Mechanistically, under normal acute activation, TCR signaling triggers a transient influx of calcium, activating the phosphatase calcineurin, which dephosphorylates NFAT (11). In this scenario, NFAT enters the nucleus and partners with the transcription factor AP-1 to initiate effector gene programs (such as the production of IL-2 and IFN-γ). However, during chronic stimulation, the persistent calcium influx keeps NFAT localized within the nucleus for extended periods (43). Crucially, in the context of exhaustion, NFAT often acts without its partner AP-1. This “partnerless” NFAT activity shifts the transcriptional focus away from effector function and toward the *de novo* induction of TOX. Once induced, TOX operates in a calcineurin-independent feed-forward loop, sustaining its own expression independently of ongoing antigen stimulation (**Table 2**, **Figure 2**). This feedback mechanism effectively “commits” the T cell to the exhaustion trajectory. It signals the end of the effector phase and the gradual establishment of a new developmental state characterized by high inhibitory receptors expression and altered metabolism. Indeed, within the population of exhausted CD8^+^ T cells, a subgroup of more responsive progenitor exhausted T cells (T_pex_) can be distinguished by the terminally exhausted T cells (T_tex_), which represent the ultimately differentiated state of the exhaustion program (44, 45).

**Figure 2:**
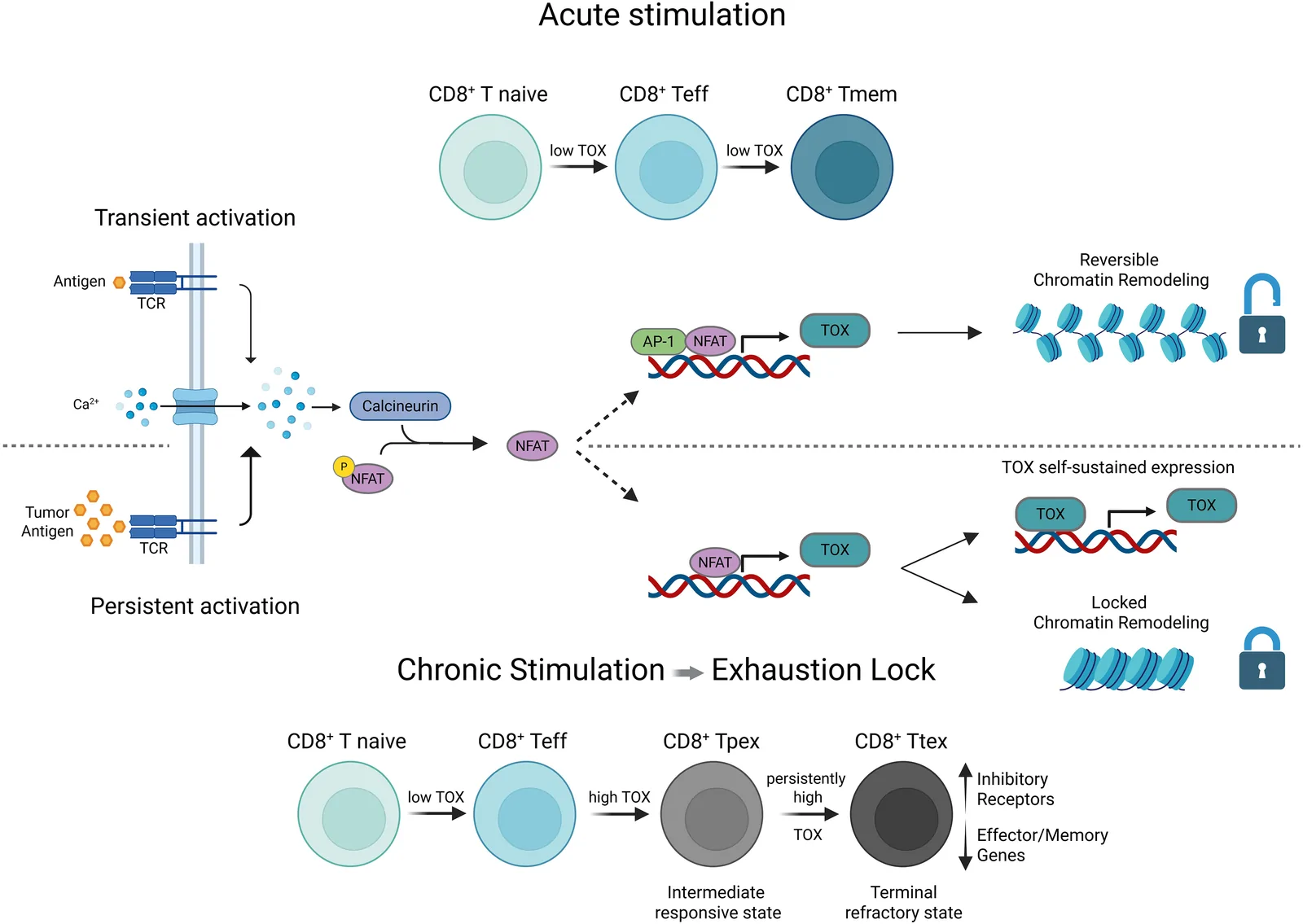
Role of TOX in CD8^+^ T cell exhaustion. Schematic representation of TOX activation and function during acute stimulation (upper half) and chronic stimulation (lower half). For details see text. CD8^+^, CD8^+^ T cell; Teff, effector T cell; Tmem, memory T cell; Tpex, progenitor exhausted T cell; Ttex, terminally exhausted T cell. See text for details. (Image generated with BioRender).

**Table 2:** Mechanistic features of T cell maturation and of T cell exhaustion during acute and chronic stimulation.

Feature	Acute Stimulation (infection)	Chronic Stimulation (tumor)
Antigen Presence	Transient	Persistent
NFAT Behavior	NFAT + AP-1 (Cooperative)	NFAT (Partnerless)
TOX Levels	Low	High
Epigenetic State	Flexible/Memory Potential	Remodeled/”Locked”
Functional Outcome	Effector & Memory Formation	T cell Exhaustion (Tex)

Accordingly, in human melanoma and non-small cell lung cancer (NSCLC), TOX levels tend to increase as intra-tumoral CD8^+^ T cells become more exhausted, and TOX knockdown in CD8^+^ T cells resulted in downregulation of PD-1, TIM3, TIGIT and CTLA-4 (46). These results suggest that TOX promotes intra-tumoral T cell exhaustion by upregulating immune checkpoint proteins in cancer. Moreover, while T_eff_ and T_mem_ can form without TOX, its expression is necessary and sufficient for exhausted T cell development (11). Indeed, TOX^Flox/Flox^ CD4^Cre^ (cKO) P14 cells when injected in mice were not intrinsically unable to form CD8^+^ T cells that could persist following acute infection, including T_mem_, but rather had a specific defect in the generation of exhausted T cells (T_ex_, 11). Furthermore, in hepatocellular carcinoma TOX reduces PD1 degradation and promotes PD1 translocation to the cell surface in CD8^+^ T cells, thus maintaining high PD1 expression at the cell surface (47). The downregulation of TOX expression in CD8^+^ T cells exerts synergistic effects with anti-PD1 therapy, highlighting a promising strategy for cancer immunotherapy (47).

### 5.2 An epigenetic point of no return?

This “lock” is not merely a change in gene expression but a fundamental alteration of the epigenetic landscape. TOX-mediated remodeling contributes to a progressively stabilized and consolidated exhaustion program rather than a purely transient state. This helps explain why simply blocking PD-1/PD-L1 pathways often fails to fully “re-program” these cells; the underlying DNA architecture remains physically constrained by the TOX-driven epigenetic signature, keeping the T cell killing potential under sustained molecular arrest. TOX belongs to the HMG-box family, which binds DNA in a sequence-independent but structure-dependent manner (4). By distorting the physical structure of the double helix, TOX allows other transcription factors to access regions of the genome that were previously “closed” or bundled tightly. Accordingly, TOX creates new Open Chromatin Regions (OCRs) at loci encoding inhibitory receptors (PD-1/PDCD1, TIM-3/HAVCR2, LAG-3, TIGIT, CD244), while closes chromatin at effector/memory gene loci, including Klrg1, Gzma, Gzmb, Gzmm, Clnk, Zeb2, and Nr4a1 (48). Deletion of TOX in tumor-specific T cells prevents upregulation of inhibitory receptors and keeps their chromatin largely inaccessible (48). Notably, the epigenetic changes caused by TOX corresponded to functionally relevant events since there was a strong association of chromatin opening with increased gene expression and *vice versa* (11), indicating a role for TOX in both opening and closing genomic regions associated with T_ex_ or T_eff_ differentiation, respectively. Of note, one of the most critical epigenetic roles of TOX is its ability to regulate its own expression. Persistent NFAT (without AP-1) triggers the initial production of TOX (43), which then binds to its own promoter/enhancer regions creating a positive feedback loop that maintains high TOX levels, and therefore the exhaustion program active, independently of the original TCR signal (11) (**Figure 2**). However, this stabilization is not uniform across the T_ex_ compartment: progenitor and intermediate T_ex_ populations retain greater plasticity and can undergo reversible transitions, whereas progression toward terminal exhaustion is associated with more extensive epigenetic remodeling and reduced reversibility (49). Thus, antigen removal does not necessarily erase the epigenetic imprint of exhaustion, although the extent of functional and epigenetic recovery may depend on the differentiation state of the T_ex_ population (50, 51). Checkpoint blockade (e.g., anti-PD-1) only transiently improves T_ex_ function without altering the underlying epigenetic landscape, because TOX-enforced chromatin states remain intact (52). Moreover, TOX is required to maintain chromatin accessibility and transcriptional patterns even in committed T_ex_ cells, not just during initiation. Induced TOX ablation in established T_ex_ cells reduces expression of inhibitory receptors and results in apoptotic loss of T_ex_ cells and partial reprogramming toward effector-like states (38).

## 6 TOX expression in hematologic vs. solid malignancies: beyond immune regulation

Pan-cancer TCGA analysis shows that TOX expression is closely associated with immune cell infiltration, immune checkpoint expression, and prognosis across cancer types (53). The biological significance of the TOX protein extends far beyond its established role in orchestrating T cell-exhaustion, acting as a multifaceted novel oncogene in various contexts (**Table 3**).

**Table 3:** Pathological expression patterns and functions of TOX paralogs subdivided according to type of cancer (hematological and solid tumors) and cell-specific context (tumor cells and immune infiltrating cells) within the tumor.

Cancer	Paralogs	Tumor cells	Immune cells
Hematological tumors			
T-ALL	TOX, TOX2	-Suppression of apoptosis -Promotion of proliferation -Promotion of cell cycle dysregulation	-T cell exhaustion
AML	TOX, TOX2, TOX3	-Restriction of apoptosis, -Promotion of proliferation -Higher TOX, TOX2 and TOX3 correlate with poor OS	-T cell exhaustion
MM	TOX		-Immunosuppression
cHL	TOX, TOX2		-T cell exhaustion
NKTL	TOX2	-Promotion of proliferation survival and metastasis -High TOX2 correlates with worse survival	
Solid tumors			
Pancreatic	TOX		-T cell exhaustion
Colorectal	TOX	-Tumor suppressor, restriction of proliferation, migration and invasion -Support of CD8+ T cell cytotoxicity -High TOX associated with better OS and MSI status; low TOX with advanced TNM stage and poor outcome	-CD8+ T cells: master regulator of exhaustion -CD4+ T cells: required for Th1 effector function, promotes IFN-γ production and antitumor immunity -High TOX in infiltrating immune cells correlates with a high ImmunoScore
	TOX3	-Promotion of proliferation -Inhibition of apoptosis -High TOX3 correlates with shorter DFS	
NSCLC	TOX		-T cell exhaustion
LUAD	TOX	-TOX downregulated relative to normal tissue -Lower TOX correlates with poor prognosis	-T cell exhaustion -Stem-like TCF1+CD8+ T cells (TOX-low) associate with improved DFS and OS
	TOX4	-Promotion of cell migration and invasion -High TOX4 correlates with shorter OS	
Breast	TOX		-T cell exhaustion -Correlates with T cell infiltration, exhaustion markers and inflammation in all subtypes -High TOX associated with improved outcome (Luminal B and TNBC) -Higher TOX in tumor stroma associated with improved pathological response to neoadjuvant chemotherapy and longer DFS and OS
	TOX3	-Promotion of cancer progression (luminal)	
Neuroblastoma	TOX		-T cell exhaustion
Cervical	TOX		-Promotion of immune tolerance through ferroptosis -High TOX in TILs correlates with shorter DFS and OS
Others	TOX		-T cell exhaustion

See details in text.

### 6.1 Hematologic cancers

In hematologic malignancies, TOX plays dual roles (54): (1) as a driver of T cell exhaustion within the tumor immune microenvironment, and (2) as a direct oncogenic factor in certain lymphoid neoplasms. Indeed, in some lymphomas and T-cell Acute Lymphoblastic Leukemia (T-ALL), TOX and its paralog TOX2 are frequently hijacked by the cancer cells themselves to drive suppression of apoptosis, promotion of cell proliferation, and cell cycle dysregulation, independently of their immunological functions (7). In T-ALL cell lines, nuclear TOX2 forms a complex with LCOR and HDAC3 to repress TIM-3, promoting leukemogenesis. TOX2 nuclear-cytosol translocation is regulated by Sirt1/TBK1-mediated deacetylation. Knockdown of TOX, TOX2 or LCOR induces apoptosis in T-ALL cells (16). Small molecule inhibitors targeting the TOX protein-protein interactions (PPIs) have been identified as an emerging therapeutic approach for treating T cell exhaustion and leukemia. A promising small molecule, KI-TOX-A3, disrupts TOX PPIs, leading to TOX degradation, showing selective cytotoxicity in T-ALL cells and reversing T cell exhaustion in CD8^+^ T cells (55).

Similarly, in Acute Myeloid Leukemia (AML), high TOX expression promotes tumor cell proliferation and restricts apoptosis, with TOX knockdown inducing G0/G1 arrest and reducing tumor burden *in vivo*, partly through activation of the IFN-α/IRF7 signaling axis (56). Moreover, TOX expression is significantly elevated in CD3^+^, CD4^+^, and CD8^+^ T cells from *de novo* AML patients and co-expressed with exhaustion markers PD-1, TIM-3, and CD244. This exhaustion phenotype is partially reversed upon complete remission after chemotherapy (57). Higher TOX, TOX2, and TOX3 expression in AML patients correlates with poor overall survival (58). In both peripheral blood and bone marrow of Multiple Myeloma (MM) patients, TOX^+^ T cells, including regulatory T cells (Tregs), co-expressing PD-1, TIM-3, and CD244 are significantly increased, suggesting a broad immunosuppressive landscape driven by TOX (59).

Veldman et al. found that the dominant CD4^+^CD26^-^ T cell population surrounding tumor cells in classical Hodgkin Lymphoma (cHL) expressed TOX and TOX2, along with immune checkpoints (PD-1, LAG-3) and chemokines. Importantly, these cells were antigen-experienced but polyclonal and exhausted, likely explaining the very high response rates to checkpoint inhibitors in cHL (60).

In extranodal NK/T-cell lymphoma (NKTL), the TOX family member TOX2 is driven by super-enhancer activation, is aberrantly overexpressed relative to normal NK cells, and acts as a bona fide oncogene, promoting proliferation, survival, and metastasis via the downstream effector phosphatase of regenerating liver-3 (PRL-3). High TOX2 expression correlates with worse survival, and TOX2 knockdown impairs cell proliferation, survival, and tumor formation *in vivo* (17).

### 6.2 Solid cancers

In many solid tumors, TOX remains a hallmark of CD8^+^ T cell exhaustion, where it transcriptionally upregulates checkpoints such as PD-1 and LAG-3, thereby facilitating immune evasion. This exhaustion axis has been documented across multiple solid tumor contexts, including pancreatic cancer (61) and ovarian cancer (62), and is further reinforced by pan-cancer analyses demonstrating that TOX expression correlates with T cell-exhaustion signatures and immune checkpoint co-expression across 30 tumor types (63).

#### 6.2.1 Pancreatic cancer

In pancreatic ductal adenocarcinoma (PDAC), T cell exhaustion is a major mechanism of immune evasion. Exhausted tumor-infiltrating lymphocytes (TILs) in PDAC show high co-expression of inhibitory receptors (PD-1, TIM-3, LAG-3, CTLA-4, TIGIT), all of which are upregulated downstream of TOX (61). In a murine pancreatic cancer model, IL-18 receptor signaling was shown to induce CD8^+^ T cell exhaustion partly via upregulation of TOX and Eomes, and NLRP3-mediated IL-18 signaling correlated with shorter survival in TCGA pancreatic carcinoma patients (64).

#### 6.2.2 Colorectal cancer

In colorectal cancer (CRC), the role of TOX is context-dependent. Indeed, recent research has revealed that TOX also acts directly as a tumor suppressor within the CRC cells themselves, and its expression tends to be downregulated as CRC progresses (65). Moreover, TOX inhibits the mTOR signaling pathway, which is a primary driver of cell growth, therefore restricting the proliferation, migration, and invasion of cancer cells. High TOX expression in tumor tissues is associated with better overall survival (OS) and MSI status. Conversely, low TOX expression correlates with more advanced TNM stages and poor clinical outcomes in CRC (65). Authors showed that TOX-high tumor cells express lower levels of the methionine transporter SLC43A2, reducing their methionine uptake. This reduced methionine competition with CD8^+^ T cells supports CD8^+^ T cell cytotoxicity and decreases apoptosis (66). Additionally, there is a known negative correlation between TOX and the *Fusobacterium nucleatum*, a pathogen often linked to CRC progression, and which appears to suppress TOX expression to help the tumor evade the immune system (67). In contrast to TOX, high levels of its paralog TOX3 in CRC tissues correlate with a shorter disease-free survival (68). In this study TOX3 was shown to promote proliferation and inhibits apoptosis in CRC cell lines through the MAPK signaling pathway and to promote cancer growth upon grafting in mouse models (68).

Within the immune cells surrounding the tumor, TOX plays a vastly different role from that played in the context of tumor cells. While in CD8^+^ cytotoxic T cells TOX is a master regulator of exhaustion, in CD4^+^ T cells TOX appears to be beneficial. Recent studies suggest that TOX is required for the effector function of Type 1 Helper cells, promoting the production of IFN-γ and enhancing anti-tumor immunity (12). Moreover, high levels of TOX in the tumor microenvironment (specifically in infiltrating immune cells) often correlate with a high ImmunoScore, suggesting these patients may be better candidates for immune checkpoint inhibitors (65).

A recent study by Kim et al., points out a VEGF-A induced TOX signaling cascade, which drives transcriptional reprogramming for T-cell exhaustion in anti-PD-1 resistant colorectal cancer (69). In the presence of VEGF-A, anti-CD3 stimulated T cells from healthy human subjects presented upregulation in inhibitory receptors and the expression of proapoptotic molecules, indicating the critical role of VEGF-A in TOX-regulated epigenetic changes in chromatin of T cells that result in attenuated T cell effector capacity (70).

#### 6.2.3 Lung cancer

TOX expression increases progressively as CD8^+^ T cells transition from progenitor to terminally exhausted states in non-small cell lung cancer (NSCLC) tumors, and was identified as the only transcription factor consistently upregulated in exhausted (PD-1-high) tumor-infiltrating CD8^+^ T cells across both melanoma and NSCLC in single-cell transcriptome analyses. Accordingly, TOX expression in TILs is highly predictive of overall survival and anti-PD-1 efficacy in NSCLC patients (46).

A dedicated lung adenocarcinoma (LUAD) study using TIMER, Kaplan-Meier Plotter, GEPIA2, and single-cell RNA-seq showed that TOX was downregulated in LUAD relative to normal tissue, and lower TOX expression correlates with poor prognosis, with particular prognostic significance in early-stage LUAD, ever-smokers, and low-TMB patients. Moreover, higher TOX expression positively correlates with increased immune infiltration of CD8^+^ T cells, CD4^+^ T cells, mast cells, and functional T cells, and single-cell RNA-seq confirms TOX enrichment in exhausted CD4^+^ and CD8^+^ T cell populations, co-expressed with key exhaustion markers (PD-1, TIM-3, TIGIT, and CXCL13, 71). A separate study in LUAD (n=191, stages II–III) using multiplex fluorescence immunohistochemistry found that higher TCF1^+^CD8^+^ T cell infiltration (stem-like, TOX-low) was associated with improved DFS and OS, and that in tumor-draining lymph nodes invaded by tumor, the proportion of stem-like TCF1^+^CD8^+^ T cells was significantly decreased, consistent with TOX-driven terminal exhaustion at sites of chronic antigen exposure (72). On the other hand, high expression of TOX4 in lung adenocarcinoma tumor tissues correlates with shorter overall survival of patients; an evidence very likely associated with a role of TOX4 in promoting migration and invasion of lung cancer cells (73).

#### 6.2.4 Breast cancer

The role of TOX is highly cancer-type dependent, often exhibiting a tissue-specific divergence most evident in the breast cancer paradox. In luminal breast cancer, high expression of TOX3 has been shown to regulate estrogen receptor (ER) target genes, such as *TFF1* and *CXCR4*, driving cancer progression even in the absence of estrogen (20). However, recent data highlights a prognostic shift based on the cellular source: while TOX expression in tumor cells often correlates with poor outcomes, its high expression in tumor-infiltrating immune cells may paradoxically signal a better prognosis in certain subtypes. A comprehensive analysis of TCGA and GEO breast cancer datasets across all four intrinsic subtypes clarified this cell-of-origin distinction (74). TOX expression was found to be almost exclusively contributed by tumor-infiltrating immune cells rather than by malignant epithelial cells, which showed weak or absent TOX staining. Notably, the basal-like (triple-negative) subtype displayed the highest overall TOX expression among the four subtypes and was the only subtype in which TOX was elevated in tumor tissue relative to adjacent normal breast tissue, consistent with the greater baseline immune infiltration and immunogenicity that characterizes triple-negative breast cancer (75). Across all subtypes, TOX expression correlated positively with T cell infiltration, T cell exhaustion markers, and markers of active inflammation. In terms of clinical outcome, high TOX expression was associated with improved overall survival, relapse-free survival, and distant-metastasis-free survival overall, with the prognostic benefit being strongest in the luminal-B subtype; in basal-like tumors specifically, high TOX was most strongly associated with improved distant-metastasis-free survival (74). This suggests that in these breast cancer contexts, TOX-positive immune cells represent an active, though struggling, inflammatory response, transforming a “cold” tumor into an “immunologically warm” one and correlating with improved clinical outcomes. This interpretation is further supported by two independent studies demonstrating that higher TOX expression in the tumor stroma is associated with an improved pathological response to neoadjuvant chemotherapy in locally advanced breast cancer (76), and with longer disease-free and overall survival (77), reinforcing the concept that TOX-positive infiltrates mark a more immunologically engaged and treatment-responsive tumor microenvironment. Ultimately, TOX serve as a critical regulator of cell apoptosis, growth, metastasis, and DNA repair, but its final impact on patient survival is dictated by whether it is acting as an intrinsic oncogenic driver within malignant cells or an extrinsic marker of the exhausted, yet immunologically engaged, tumor-immune landscape.

#### 6.2.5 Other cancers

In neuroblastoma patients, T cell exhaustion was demonstrated to correlate with the elevated expression of TOX in freshly sorted CD3^+^ T cells as well as in anti-CD3 stimulated PBMCs (78). This observation supports the idea that the hypomethylation of the TOX promoter may be one of the initiators that regulates TOX expression and enables TOX to play a crucial role in T cell exhaustion reprogramming in neuroblastoma patients.

In the context of cervical cancer, increased expression of TOX in tumor-infiltrating lymphocytes was shown to correlate with shorter disease-free survival and overall survival (79). This recent work presented a new role of TOX, which in concert with STING and in response to enriched levels of tumor-derived lactate, triggers ferroptosis in tumor-infiltrating CD8^+^ T cells in mouse models, therefore promoting immune tolerance (79).

## 7 Therapeutic horizons

### 7.1 TOX and immunotherapy

Following on the established role of TOX in physiological lymphopoiesis, an emerging role as a master regulator of CD8^+^ T cell exhaustion and its context-dependent behavior across malignancies make TOX a crucial determinant of responses to immune checkpoint blockade.

The findings so far described suggest that TOX expression in CD4^+^ T cells may carry prognostic and predictive value distinct from, and potentially complementary to, its established role in CD8^+^ T cells. TOX has been identified as a master transcription factor of T cell exhaustion with important ramifications for responses to PD-1/PD-L1 blockade. Numerous studies have confirmed an association between TOX expression and the epigenetic and transcriptional programming of exhausted CD8^+^ T cells, those that are constitutively expressing inhibitory receptors and display reduced effector function (11). TOX in this context is not simply a marker but rather, an initiator of the exhausted phenotype. Crucially, the association between TOX levels and immunotherapy outcomes seems to be less than simple. TOX^+^ T cells comprise the dominant PD-1/PD-L1 target within tumor-infiltrating lymphocytes (80, 81). Indeed, TOX^+^ T_ex_ cells do contain a subset with proliferative capacity (T_pex_) that can be reinvigorated by checkpoint blockade and is associated with therapeutic responses. Conversely, T_tex_ cells that show pronounced as well as persistent TOX expression are epigenetically fixed and largely incapable of functional re-invigoration, even following PD-1 blockade (44, 45).

Thus, high TOX expression could represent multiple biologically different states; reversibly exhausted, progenitor/intermediate populations that are still responsive to immunotherapy and a terminally differentiated population that is largely refractory (**Figure 2**). Therefore, TOX alone may not have enough predictive power to be a viable biomarker unless complemented with additional markers of these distinct subsets (such as e.g. high TCF1 expression or chromatin accessibility profile). Yet properly contextualized to the cell and molecular ball game, TOX expression may prove useful as a biomarker for identifying patients who are likely to respond or not respond to PD-1/PD-L1 blockade (82). In summary, instead of suggesting ultimate irreversibility of TOX-expressing T cells, this indicates a different level of exhaustion where possibilities for successful activation differ based on the differentiation stage of the TOX^+^ T cell compartment. Within this frame, combinatorial strategies seem especially promising. Combined blockade of PD-1 and TIGIT partially rescues TOX-expressing terminally exhausted CD8^+^ tumor-infiltrating lymphocytes (81), further supporting the rationale for a combination of checkpoint therapies with function on multiple inhibitory pathways.

### 7.2 TOX as a promising therapeutic target

TOX represents a promising, but challenging, target for therapeutic intervention due to its key role in the establishment and maintenance of T cell exhaustion. Ideally, suppression of TOX or inhibition of its downstream programs in CD8^+^ T cells would prevent these cells to enter the exhaustion state and allow them to differentiate into functional effector cells with consequent increase in anti-tumor immunity. Nonetheless, there are risks to targeting TOX therapeutically due to its involvement both in immune protection mechanisms and in promoting cancer progression. It has been suggested that TOX-mediated exhaustion functions as a protective mechanism against excessive immune activation and immunopathology (83). It has been further described that TOX actively contribute to ferroptosis in TILs (79). Therefore, tipping this regulatory axis would be predicted to permit uncontrolled activation of T cells and enhance the propensity to autoimmunity or tissue injury (38).

Different attempts to reduce TOX function have been proposed and already tested in preclinical settings. The small molecule KI-TOX-A3 was shown to inhibit TOX protein-protein interaction and target TOX to proteasome-mediated degradation (55). Another way to achieve TOX downregulation is based on the use of siRNAs against TOX transcripts (84). Taking into account the pleiotropy of TOX physiological functions, a global inhibition of TOX might trigger autoimmunity or compromise host protective immunity. This is the reason why cell type-specific targeting strategies should be preferred to an approach based on a pan-TOX suppression. Strategies targeting TOX-driven transcriptional and epigenetic programs might enhance the efficacy not only of immunotherapy beyond checkpoint inhibition, but also of adoptive cell therapies such as CAR-T cells (85) that still poorly perform in solid tumors, due to dysfunction associated with exhaustion. In light of the studies on TOX-deficient CAR-T cells (13), the possibility to modulate in a controlled manner TOX expression or activity during T cell engineering (86) may help preserving a less exhausted and more responsive state, thus enhancing persistence and cytotoxic potential *in vivo*. In this context, recent evidence indicates that the functions of TOX family members in CAR-T cells are more complex than previously appreciated. Collins and colleagues demonstrated that TOX and TOX2 exert distinct and non-redundant roles in human CAR-T cells: whereas TOX knockdown increased the proportion of central memory T cells (T_cm_), TOX2 proved necessary for optimal T_cm_ differentiation and proliferation (87). However, excessive TOX2 expression also promoted transcriptional programs associated with exhaustion precursor cells, indicating that both insufficient and excessive TOX2 activity may be detrimental (87). These findings suggest that therapeutic strategies should not simply aim to abolish TOX family activity but rather fine-tune the expression or function of individual family members during CAR-T cell engineering, thereby maximizing persistence and memory formation while limiting the acquisition of exhausted states. This perspective is further supported by recent advances in epigenetic engineering of CAR-T cells. Ahn and colleagues highlighted that epigenetic regulators can critically influence the balance between T cell-exhaustion, persistence and memory differentiation (88). In particular, disruption of TET2 in CAR-T cells has been associated with an increased proportion of central memory-like cells, enhanced proliferative capacity and improved cytotoxic potential (88). These observations reinforce the concept that therapeutic manipulation of exhaustion-associated transcriptional and epigenetic networks may enhance CAR-T cell fitness. Importantly, given the close functional interplay between epigenetic regulators and exhaustion-associated transcription factors such as TOX, future strategies may benefit from simultaneously considering the TOX axis and broader chromatin-remodeling pathways to promote durable CAR-T cell responses.

## 8 Discussion

The pleiotropy of effects mediated by TOX proteins reflects the profound changes that this family of chromatin modulators performs at a molecular level. The multiple outcomes induced by TOX rely upon the sequence-independent/structure-dependent binding of chromatin and the participation of this family of proteins in a bigger chromatin remodeling complex to which - dependently on the context - specific cofactors are recruited in order to regulate the transcriptional expression of different genes associated with tissue-specific function (12, 89). TOX-mediated impact on epigenetics is decisive and suitably engaged in several different crucial steps during lymphopoietic development as well as during immune response, but also meanly reactivated to support tumor progression. As a result of the concomitant role of TOX as immunomodulator and oncogene, future approaches seeking to restore anti-tumor function while preserving immune homeostasis will need to fine-tune TOX activity rather than extinguish it entirely – and possibly address this modulation to the specific cellular subtypes of interest. Notwithstanding, TOX proteins represent ideal prognostic biomarkers for many different cancer types - especially if suitably combined to other indicators of the tumor environment - even knowing the opposite effects of TOX in some cancer typologies and within the same cancer type among different cell contexts. Whether a combined score integrating TOX expression across both the CD4^+^ and the CD8^+^ T cell-compartments could refine its utility as a biomarker remains, however, an open question for future studies. Beyond its potential as a biomarker, the context-dependent activity of TOX has also important implications for adoptive cell therapies. Recent findings in CAR-T cells further support the context-dependent role of TOX-family members in regulating T cell differentiation and persistence. These observations suggest that precise modulation of TOX-related transcriptional programs, rather than complete inhibition of TOX activity may represent a promising strategy to improve the durability of adoptive cell therapies.

## Glossary

TOX: Thymocyte Selection-Associated High-Mobility Group Box Protein
TIL: Tumor-Infiltrating Lymphocyte(s)
TME: Tumor Microenvironment
TCR: T-Cell Receptor
ICB: Immune Checkpoint Blockade
HMG: High-Mobility Group
T-ALL: T-cell Acute Lymphoblastic Leukemia
NHEJ: Non-Homologous End Joining
NK: Natural Killer
LTi: Lymphoid Tissue inducer
ILC: Innate Lymphoid Cell
NKp: NK progenitor
Mst1: Mammalian Sterile 20-like Kinase 1
ChIP-seq: Chromatin Immunoprecipitation sequencing
CLP: Common Lymphoid Progenitor
DN: Double Negative
DP: Double Positive
NFAT: Nuclear Factor of Activated T Cells
SP: Single Positive
ThPOK: T-helper-inducing POZ/Kruppel-like factor
Id2: Inhibitor of DNA Binding 2
FOXO1: Forkhead Box O1
GATA3: GATA Binding Protein 3
T-bet: T-box expressed in T cells
ETS: E26 Transformation-Specific
IFN-γ: Interferon-gamma
MHC: Major Histocompatibility Complex
CXCL13: C-X-C motif Chemokine Ligand 13
AICD: Activation-Induced Cell Death
Teff: Effector T cell
Tmem: Memory T cell
Tpex: Progenitor exhausted T cell
Ttex: Terminally exhausted T cell
NSCLC: Non-Small Cell Lung Cancer
Tex: Exhausted T cell
Flox: Flanked by loxP
cKO: Conditional Knockout
PD-1: Programmed Cell Death Protein 1
TIM-3: T-cell Immunoglobulin and Mucin-domain containing-3
TIGIT: T cell Immunoreceptor with Ig and ITIM domains
CTLA-4: Cytotoxic T-Lymphocyte-Associated Protein 4
OCR: Open Chromatin Region
LAG-3: Lymphocyte-Activation Gene 3
TCGA: The Cancer Genome Atlas
PPI/PPIs: Protein-Protein Interaction(s)
LCOR: Ligand-Dependent Corepressor
HDAC3: Histone Deacetylase 3
CREB–CBP: cAMP Response Element-Binding Protein–CREB-Binding Protein
GWAS: Genome-Wide Association Study
DNA-PK: DNA-Dependent Protein Kinase
PNUTS: Phosphatase Nuclear Targeting Subunit
AML: Acute Myeloid Leukemia
MM: Multiple Myeloma
Treg/Tregs: Regulatory T cell(s)
cHL: Classical Hodgkin Lymphoma
NKTL: NK/T-cell Lymphoma
PRL-3: Phosphatase of Regenerating Liver-3
PDAC: Pancreatic Ductal Adenocarcinoma
CRC: Colorectal Cancer
OS: Overall Survival
MSI: Microsatellite Instability
TNM: Tumor, Node, Metastasis
LUAD: Lung Adenocarcinoma
TCF1: T-cell Factor 1
ER: Estrogen Receptor
PBMCs: Peripheral Blood Mononuclear Cells
siRNAs: Small Interfering RNAs
Tcm: Central Memory T Cell(s)
DFS: Disease-Free Survival
TMB: Tumor Mutational Burden
CAR-T: Chimeric Antigen Receptor T cell(s)
VEGF-A: Vascular Endothelial Growth Factor A
STING: Stimulator of Interferon Genes
Sirt1: Sirtuin 1
TBK1: TANK-Binding Kinase 1
AP-1: Activator Protein 1
IL-2: Interleukin-2
IL-18: Interleukin-18
NLRP3: NLR Family Pyrin Domain Containing 3
Eomes: Eomesodermin
TFF1: Trefoil Factor 1
CXCR4: C-X-C Motif Chemokine Receptor 4
SLC43A2: Solute Carrier Family 43 Member 2
mTOR: Mechanistic Target of Rapamycin
TET2: Tet Methylcytosine Dioxygenase 2
